# Discovery of a novel integron-borne aminoglycoside resistance gene present in clinical pathogens by screening environmental bacterial communities

**DOI:** 10.1186/s40168-020-00814-z

**Published:** 2020-03-20

**Authors:** Maria-Elisabeth Böhm, Mohammad Razavi, Nachiket P. Marathe, Carl-Fredrik Flach, D. G. Joakim Larsson

**Affiliations:** 1 Centre for Antibiotic Resistance Research (CARe), University of Gothenburg, Gothenburg, Sweden; 2 Department of Infectious Diseases, Institute of Biomedicine, Sahlgrenska Academy, University of Gothenburg, Gothenburg, Sweden; 3 Institute of Marine Research (IMR), Bergen, Norway

**Keywords:** Functional metagenomics, Antibiotic resistance, Pathogens, Environment, Aminoglycosides, Integron

## Abstract

**Background:**

New antibiotic resistance determinants are generally discovered too late, long after they have irreversibly emerged in pathogens and spread widely. Early discovery of resistance genes, before or soon after their transfer to pathogens could allow more effective measures to monitor and reduce spread, and facilitate genetics-based diagnostics.

**Results:**

We modified a functional metagenomics approach followed by in silico filtering of known resistance genes to discover novel, mobilised resistance genes in class 1 integrons in wastewater-impacted environments. We identified an integron-borne gene cassette encoding a protein that conveys high-level resistance against aminoglycosides with a garosamine moiety when expressed in *E. coli*. The gene is named *gar* (garosamine-specific aminoglycoside resistance) after its specificity. It contains none of the functional domains of known aminoglycoside modifying enzymes, but bears characteristics of a kinase. By searching public databases, we found that the gene occurs in three sequenced, multi-resistant clinical isolates (two *Pseudomonas aeruginosa* and one *Luteimonas* sp.) from Italy and China, respectively, as well as in two food-borne *Salmonella enterica* isolates from the USA. In all cases, *gar* has escaped discovery until now.

**Conclusion:**

To the best of our knowledge, this is the first time a novel resistance gene, present in clinical isolates, has been discovered by exploring the environmental microbiome. The *gar* gene has spread horizontally to different species on at least three continents, further limiting treatment options for bacterial infections. Its specificity to garosamine-containing aminoglycosides may reduce the usefulness of the newest semisynthetic aminoglycoside plazomicin, which is designed to avoid common aminoglycoside resistance mechanisms. Since the gene appears to be not yet common in the clinics, the data presented here enables early surveillance and maybe even mitigation of its spread.

## Background

Mobile resistance factors accumulate in human pathogens via horizontal transfer, often from environmental bacteria or commensals [1]. To the best of our knowledge, every resistance factor that is a clinical problem today was discovered long after it irreversibly emerged and spread in pathogenic bacteria widely enough to cause treatment failures. After discovery, propagation can often be tracked in retrospect from country to country. One of the most prominent examples is the first detection of the mobile colistin resistance gene *mcr-1* in 2015 followed by documentation of its worldwide presence [2].

If we could identify new resistance threats before they spread widely or even before they appear in pathogens, actions could be taken to reduce the risks of their emergence, spread and clinical consequences. Such actions include targeted monitoring, confinement and the addition of the new genes to genetics-based diagnostics. Moreover, the development of new antibiotics can benefit from the awareness of novel resistance mechanisms.

Large-scale functional screening of metagenomic DNA from microbial communities has revealed many unknown resistance genes in various environments [3, 4]. The approach is independent of the cultivability of the indigenous bacterial host and does not rely on similarity to known genes, making it suitable for the identification of novel resistance determinants [5]. Functional metagenomics cannot, however, easily discriminate between mobile and immobile genes, the latter having less potential of transfer to and between pathogens [6]. Moreover, common resistance genes will dominate the sequenced clones, reducing the likelihood of identifying rare, novel genes.

Integrons are genetic elements that acquire, shuffle and express promoter-less gene cassettes. Although not mobile themselves, integrons are often associated with mobile elements and located on conjugative plasmids [7]. This combination makes them ideal for acquisition and spread of resistance genes to and among pathogens, particularly integrons of class 1 [8].

The aim of this study was to discover rare, novel resistance determinants that may appear in pathogens or that have already emerged in pathogens but thus far escaped identification. We explored bacterial communities from river sediments contaminated with wastewater in a country with a high use of antibiotics (India) [9] by adapting a functional metagenomics approach focusing on amplicon libraries of integron gene cassettes. Blunt-end cloning into a plasmid under the control of a constitutively active promoter allowed us to screen for resistance against different antibiotics. To overcome sensitivity limitations, we did not analyse individual resistant clones but rather amplified and sequenced inserts of all growing bacteria on the plates via long-read technologies. We then filtered out highly abundant known resistance genes in silico to identify rare, new putative resistance genes.

## Results and discussion

To identify mobilised novel resistance determinants, class 1 integron gene cassette libraries were prepared under control of the constitutively active P_*bla*_ promoter in *E. coli* DH10β pZE21-P_*bla*_. Library sizes ranged from 5.0 × 10^9^ to 1.8 × 10^10^ bp. The estimated average insert size of all libraries ranged from 600 to 1000 bp. Libraries were screened for functional resistance determinants on 13 different antibiotics at three different concentrations (Additional file 5). No resistance against tigecycline or nitrofurantoin was detected. The other antibiotics resulted in at least 15 to up to several thousand colonies on each screening plate. All colonies were scraped off and barcoded amplicons were prepared for long-read sequencing (PacBio Sequel). Amplicon sequencing resulted in 419,709 reads. Based on the retrieved barcodes, 382,332 could be assigned to the antibiotics and concentrations used to select resistant clones. We then predicted open reading frames (ORFs) and filtered out all reads containing known antibiotic resistance genes (ARGs) responsible for the respective phenotype. Known ARGs were defined as the ORFs with identity greater than 95% and coverage greater than 85% to their homologues in the ResFinder database. The remaining 147,151 reads consisted of 46,403 unique reads (11% of all reads) with an average length of 1540 bp and they included 48,562 unique predicted ORFs. Each of the selection amplicons resulted in an average of 1450 reads with no known ARGs. These were searched manually for resistance gene candidates.

To identify promising putative novel resistance genes, we used five criteria: An ORF needed to be (i) complete and (ii) highly abundant in its set of reads while (iii) not common in the other selection sets. To ensure that the ORF originated from an integron (iv) both binding sites for the primers used to amplify the gene cassettes should be present in the read. Furthermore, (v) the candidate ORF should be the only ORF present that could explain the resistance phenotype. Despite the large number of colonies growing during functional selection, only the gentamicin and β-lactam selection sets contained ORFs fulfilling all mentioned criteria (Table 1). Thus, the metagenomic DNA samples used in this study contain exceptionally high amounts of class 1 integrons carrying a multitude of antibiotic resistance genes [10, 11], but most of them were filtered out as known or close variants of known ARGs.

**Table 1:** Abundance of candidate ORFs for functional selection

		***gar***	**ORF 1**	**ORF 2**	**ORF 3**
**Predicted functional domain**		P-loop NTPase	None	partial S41-like peptidase	DUF1851
**Length [bp]**		504	816	867	438
**Abundance in selection sets**	cefotaxime	0	**416**	**18**	**25**
	chloramphenicol	8	12	0	0
	ciprofloxacin	7	5	0	0
	colistin	0	10	0	0
	ertapenem	8	1	0	**14**
	gentamicin	**1692**	1	0	2
	imipenem	26	1	0	1
	meropenem	0	17	0	**11**
	rifampicin	0	5	0	2
	sulfamethoxazole	0	2	0	0
	trimethoprim	16	1	0	0

ORFs recovered from the set of unique reads were clustered with 97% identity threshold in each selection set. The size of clusters was reported as the abundance of candidate ORFs

Using this approach, candidate ORFs with little or no resemblance to any known resistance factor were chosen for functional verification. The putative, completely novel resistance genes found in the read set selected by β-lactams did not confer resistance when synthesised and expressed in *E. coli*. It is possible that they were able to grow in close vicinity to resistant colonies during the functional selection, since β-lactamases are secreted into the periplasm and in outer membrane vesicles to inactivate β-lactam antibiotics [12–14]. Hence, they might simultaneously provide close-range protection for surrounding non-resistant bacteria, which appeared as false positives.

**Fig. 1:**
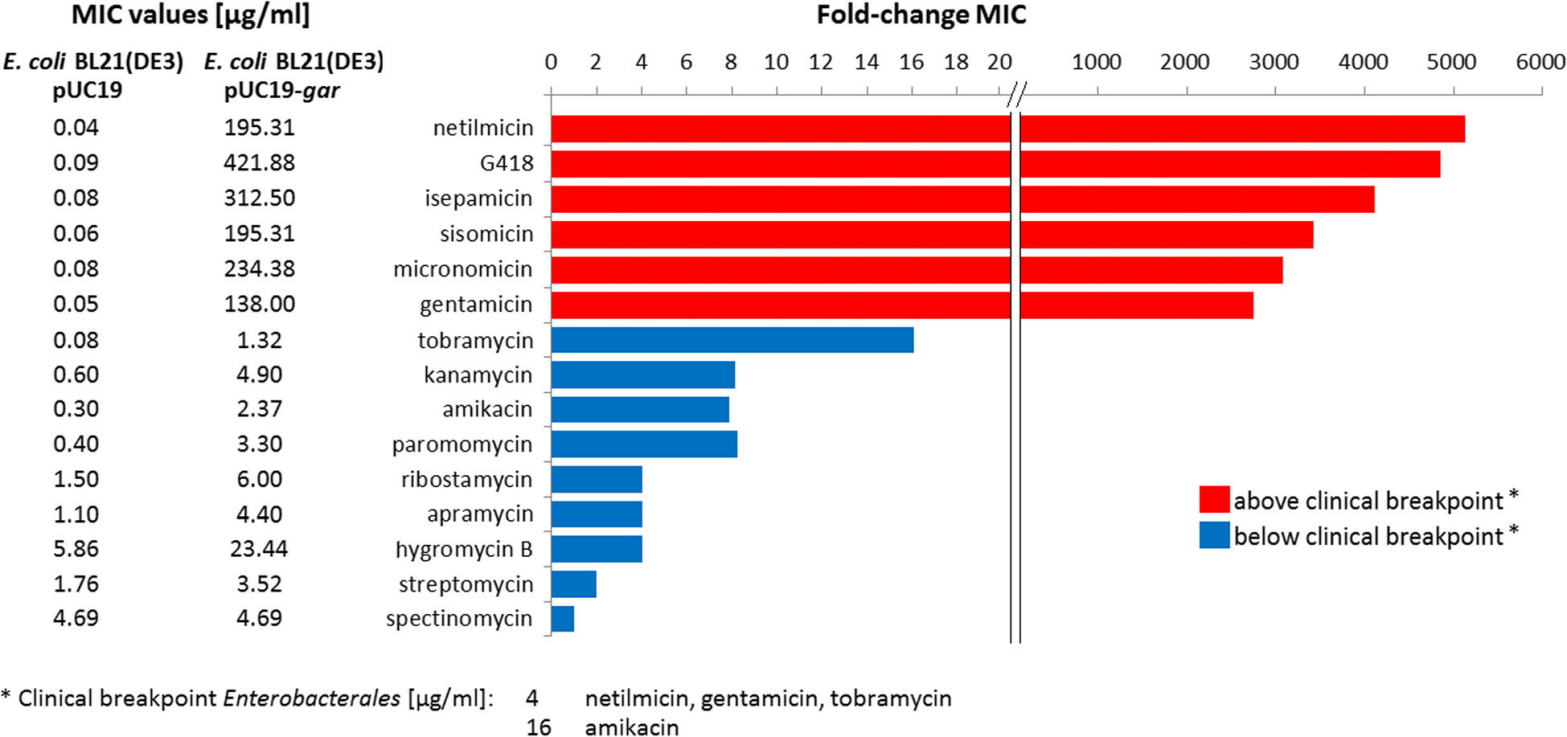
MIC values of GAR against several aminoglycosides. Molecular structures are shown in Table S2

Resistance of the candidate gene highly selected on gentamicin (designated *gar* = garosamine-specific aminoglycoside resistance) was successfully verified in *E. coli*. The protein GAR (167 aa) has an overall negative charge (pI 4.6). Since cation concentrations profoundly influenced the minimal inhibitory concentrations (MICs) (Table S1), we performed all subsequent experiments in cation-adjusted Mueller-Hinton broth. The expression of *gar* resulted in a 2760-fold MIC increase of gentamicin (Fig. 1).

Aminoglycoside modifying enzymes (AMEs) are the most clinically important resistance mechanism against aminoglycosides [15]. AMEs are divided into three enzymatic classes targeting –OH or –NH_2_ groups: aminoglycoside *N-*acetyltransferases (AACs), –*O-*nucleotidyltransferases (ANTs) and –*O-*phosphotransferases (APHs). Each class possesses a unique pattern of activity against different aminoglycosides due to their specific targets in the molecular structure [16]. We therefore tested the effectiveness of GAR against at least one representative of each aminoglycoside subclass (Fig. 1). GAR conferred resistance specifically against all six tested 4,6-disubstituted 2-deoxystreptamine–aminoglycosides containing a garosamine residue (Table S2), suggesting that it is critical for recognition.

**Fig. 2:**
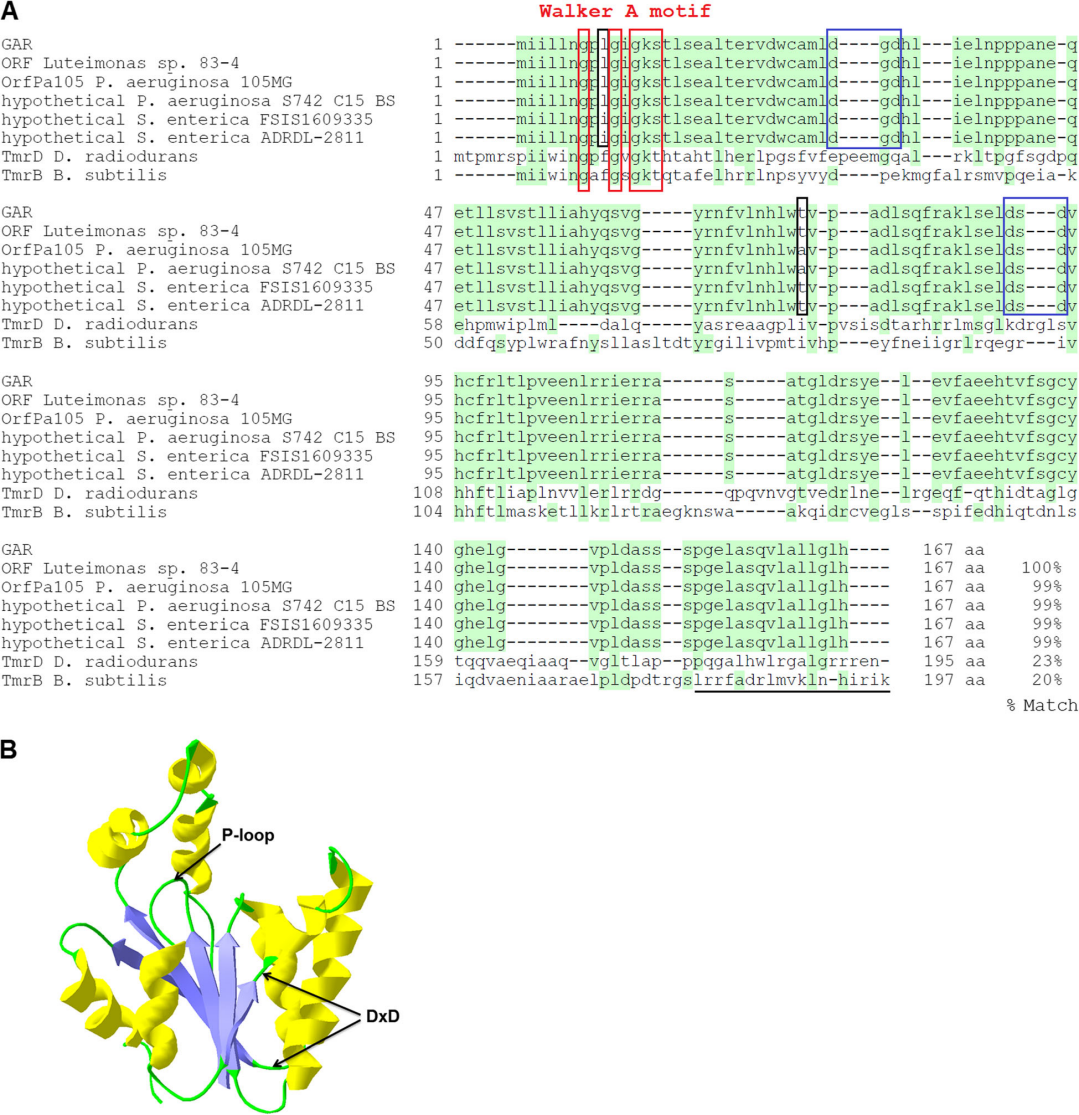
Structural motifs of GAR and related proteins. **a** Alignment of GAR with related proteins. The two amino acids that differ between GAR variants (L9I, T75A) are framed in black. The ORF from *Luteimonas* sp. 83-4 (CP029556) with 100% identity to GAR has no annotation. OrfPa105 (AJ786649) from *P. aeruginosa* 105MG and the ORF from *P. aeruginosa* S742_C15_BS without annotation (NFFO01000062.1) are identical. They differ by two nucleotides and one amino acid (T75A) from GAR. The two ORFs from *S. enterica* ADRDL-2811 (AAKHBQ010000151.1) and *S. enterica* subsp. *enterica* serovar *Johannesburg* FSIS1609335 (AAIUOI010000042.1) are identical to each other, annotated as hypothetical proteins and differ by two nucleotides and one amino acid (L9I) from GAR. Tunicamycin resistance protein TmrB as harboured by *Bacillus subtilis* (WP_003246258.1), the C-terminal membrane anchor is underlined [19], TmrD from *Deinococcus radiodurans* (WP_010888058.1) [20]. The N-terminal Walker A motif occurs in TmrB and in GAR, but the absence of the membrane anchor hints to a cytoplasmic location of GAR. The NTP-binding motif G/AXXGXGKT/S (Walker A or P-loop) is marked by red rectangles [18, 21, 22]. Searching NCBI’s conserved domain database revealed an AAA domain (**A**TPases **A**ssociated with diverse cellular **A**ctivities, pfam13238) within GAR and similarity to gluconate kinases (COG3265). GAR harbours no Walker B motif, but two potential DxD motifs, which could be responsible for Mg^2+^ binding [23], marked by blue rectangles. **b** Protein structure model of GAR. According to the structure model, the first DxD motif (aa 31–33) is located in the same cavity as the P-loop and thus more likely to participate in the NTP-binding and hydrolysis. GAR is shorter than other aminoglycoside phosphotransferases (APHs) and seems to contain five N-terminal parallel β-sheets, while APHs contain five anti-parallel β-sheets in their N-terminal domain [24, 25]

Aminoglycosides can cause mistranslation by targeting the negatively charged 16S rRNA close to the aminoacyl-tRNA site of the 30S subunit [17]. The negative charge and garosamine-specific resistance pattern make the ribosomal RNA an unlikely target for GAR. GAR contains the Walker A NTP-binding motif involved in various phosphorylation reactions [18]. It also harbours two DxD motifs (Fig. 2). Such a motif provides the ability to bind the required Mg^2+^ [18, 23, 26]. The presence of these two motifs and its negative charge strongly suggest that GAR is enzymatically active as a kinase with high affinity for positively charged targets. Aminoglycosides are indeed positively charged molecules that provide many hydroxyl residues for potential phosphorylation, including two at the garosamine moiety. One of these hydroxyl groups, located at the 4″ carbon atom, has not yet been described as a target for an AME.

**Fig. 3:**
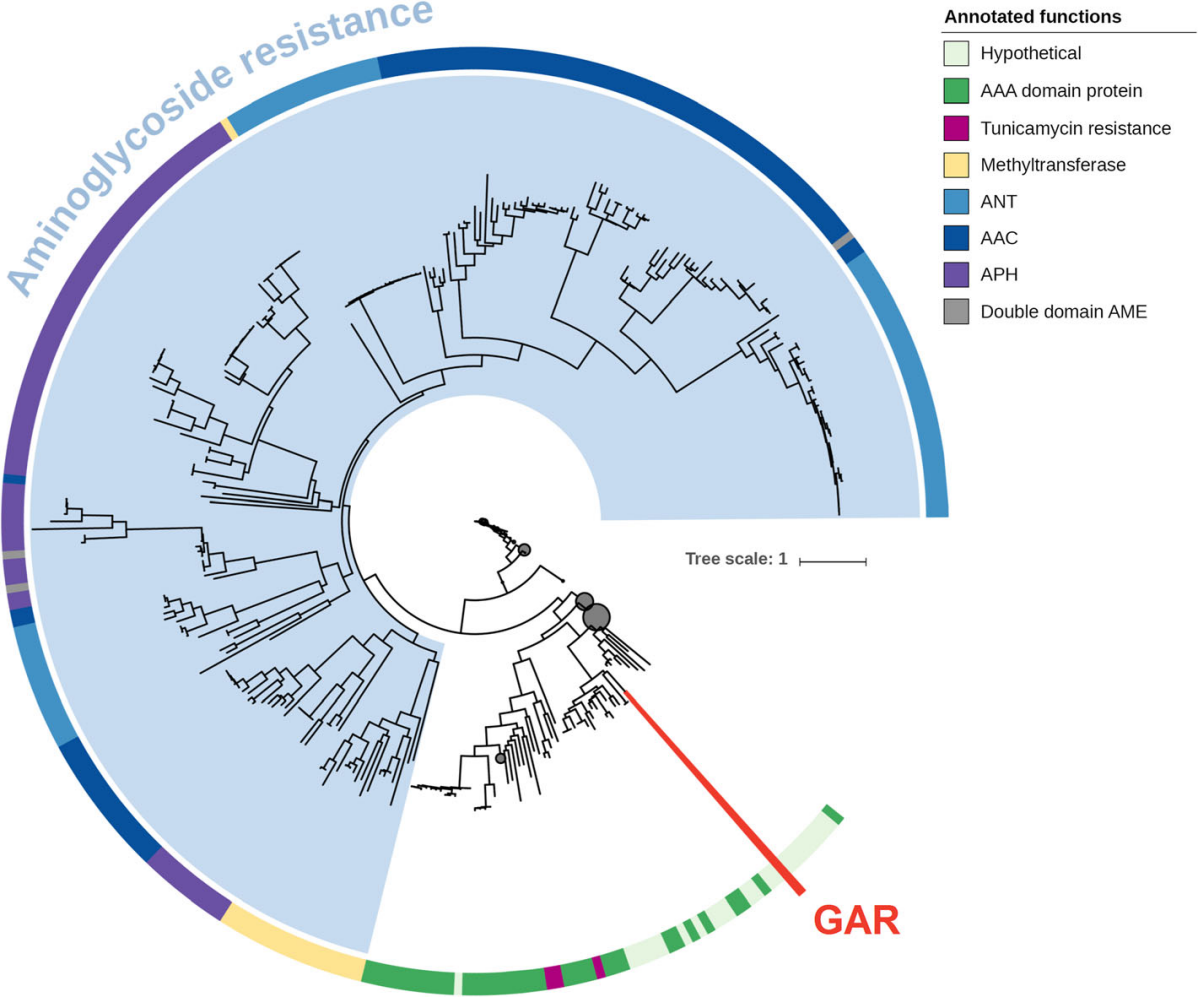
Phylogenetic tree of aminoglycoside resistance enzymes, GAR and its 1000 closest related protein sequences. Clades are collapsed (dark circles) to improve visual comparisons

There is very little similarity between GAR and all known ARGs, including AMEs (less than 20% amino acid identity). The phylogenetic tree of GAR and related proteins (Fig. 3) shows that GAR is clearly separated from the known AMEs and is located among proteins mostly annotated as hypothetical or AAA-domain containing proteins (kinases). The closest related protein from the NCBI non-redundant protein database (51% identity, WP_135484108.1) is not assigned any function. GAR shares slight amino acid sequence identity (about 20%) to tunicamycin resistance proteins (Fig. 2). Resistance to tunicamycin, a toxic nucleoside analogue produced by several *Streptomyces* species, is caused by ATP-binding membrane proteins, which also have a suspected phosphorylation activity [20, 27].

Several studies have identified a range of new ARGs in environmental samples [4, 28–32]. However, the clinical implications are less clear, since only a minor fraction of these genes are likely to end up in pathogens, e. g. due to lack of mobility, limited ecological connectivity and high fitness costs [3, 6]. Searching *gar* against the GenBank database resulted in 100% and 99% nucleotide identity with chromosomal regions of the clinical isolates *Luteimonas* sp. 83-4 (CP029556.1, China) and *P. aeruginosa* 105MG [33] (AJ786649.2, Italy), respectively. We additionally found *gar* on contigs of the clinical isolate *P. aeruginosa* S742_C15_BS (NFFO01000062.1, Italy) and on contigs of the food-borne pathogens *S. enterica* ADRDL-2811 (AAKHBQ010000151.1, USA) and *S. enterica* subsp. *enterica* serovar *Johannesburg* FSIS1609335 (AAIUOI010000042.1, USA) isolated from poultry products. In neither case was the *gar* gene recognised as a resistance gene. We can show that *gar* appears as a gene cassette in several spatially, temporally and phylogenetically separated clinical isolates, so the gene has already overcome the barrier preventing most environmental ARGs from becoming clinically relevant. Combined with its absence in related isolates (none of the other currently available *P. aeruginosa*, *Luteimonas* sp. or *S. enterica* genomes or assemblies in GenBank contain *gar*), this provides evidence that the *gar* gene is mobile. Furthermore, *P. aeruginosa* 105MG and *P. aeruginosa* S742_C15_BS belong to the exceptionally successful multidrug-resistant *P. aeruginosa* ST235 and ST111 linages, respectively, which are predominant on five continents [34]. *P. aeruginosa* frequently causes nosocomial infections with high mortality rates, especially in patients with chronic diseases, due to the accumulation of resistances against aminoglycosides, β-lactams and fluoroquinolones [34, 35]. Salmonellosis is the most important food-borne illness in the USA and the second most common in the EU [36, 37]. *Salmonella* isolates have collected a multitude of aminoglycoside resistance genes, probably due to the long-term usage of this antibiotic class in food production [38]. Occurrence in several common multidrug-resistant pathogens increases the probability for horizontal gene transfer, (co-) selection and spread of *gar*.

The structure-specific resistance pattern of AMEs implies that a single resistance gene cannot confer resistance against all aminoglycosides. However, any ARG that adds resistance to any antibiotic, which could have been a treatment possibility, reduces clinical options for treatment. For instance, *P. aeruginosa* 105MG carries four AMEs (*aph*(*3*)*-IIb*, *aac*(*6*)*-31*, *ant*(*3″*)*-Ia*, *aac*(*6*)*-Il*) plus *gar*, a combination that results in resistance against all tested aminoglycosides with the exception of apramycin (Table S3). The five AMEs target streptomycin/spectinomycin (*ant(3″*)*-Ia*); the –OH(3′) of kanamycin, G418, isepamicin, paromomycin and ribostamycin (*aph*(*3*)*-IIb*) and the –NH_2_(6′) of kanamycin, gentamicin, netilmicin, sisomicin, isepamicin, amikacin, tobramycin and ribostamycin (*aac*(*6*)*-31*, *aac*(*6*)*-Il*) [16]. Thus, even in a strain already harbouring several AMEs, *gar* extends the resistance profile (micronomicin). *Luteimonas* sp. 83-4 contains two aminoglycoside resistance genes, an *aph*(*3′*)*-XV* and *gar* (Table S4). Here, *gar* adds resistance to gentamicin, netilmicin, sisomicin and micronomicin, exacerbating already observed clinical multi-resistance. Resistance against garosamine-containing aminoglycosides is particularly worrying, since the newest semisynthetic aminoglycoside plazomicin (FDA-approved for treatment of complicated urinary tract infections [39]), which is derived from sisomicin and designed to avoid most of the common AMEs [40], includes garosamine. In the light of more and more widespread resistance to e.g. all β-lactams, an increasing dependence on aminoglycosides presenting a target for *gar* implies a growing selective pressure for bacteria to acquire, keep and spread the *gar* gene.

To examine if *gar* is present on a conjugative element in the *P. aeruginosa* 105MG isolate available to us, we attempted to transfer its aminoglycoside resistance phenotype to *E. coli* or *P. putida* via conjugation, but could detect none. Nonetheless, considering that *gar* is harboured by three distantly related species, it can obviously move horizontally. To shed light on the mechanisms involved, the genome of *P. aeruginosa* 105MG (GenBank accession: VLOE00000000) was sequenced. The *gar* gene is located within a class 1 integron on a chromosomal scaffold of 1.18 Mb. The integron is flanked by Tn*3* and Tn*21* transposon elements, which are highly abundant in bacterial genomes [41–43]. Transposable elements can promote inter-cellular mobility of integrons by insertion into a conjugative plasmid or an integrative conjugative element (ICE). The contigs upstream of the class 1 integron of the two *P. aeruginosa* isolates containing the *gar* gene cassette are identical and harbour several genes encoding conjugal transfer proteins. Hence, the entire integron could have been aquired from a conjugative plasmid/ICE, e.g. via Tn*21* [44]. There are currently 261 *P. aeruginosa* genomes in NCBI’s database of complete genomes, 74 of them (28%) contain the complete *intI1* gene (query cover and identity ≥ 97%), ten (4%) of which are plasmid-borne, but only the 105MG isolate harbours *gar*. It is thus highly plausible that *gar* has been aquired as a cassette, probably from an integron located on a yet unidentified plasmid/ICE hosted transiently by *P. aeruginosa* 105MG.

**Fig. 4:**
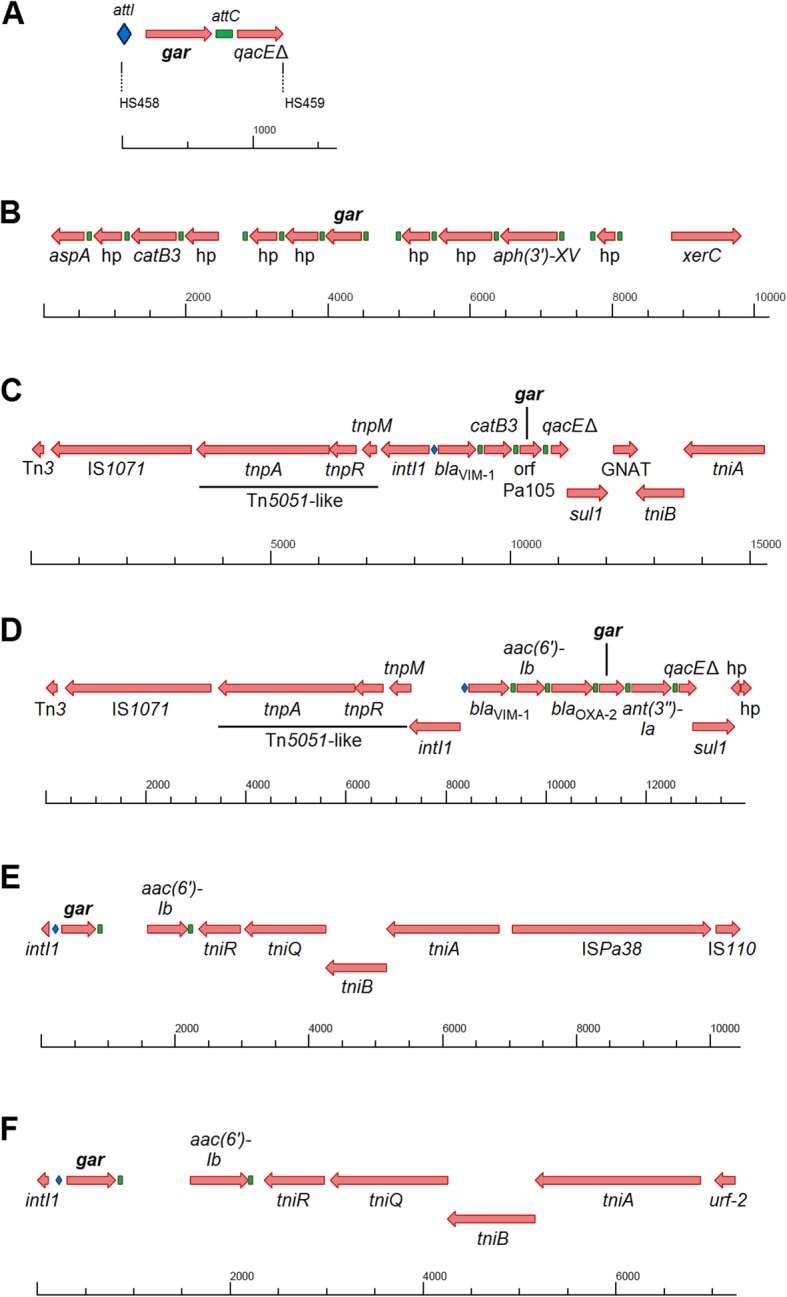
*Gar* in the six identified genetic contexts. **a** PETL metagenomics DNA sample. Primers HS458-HS459 amplified the region between *attI* site and *qacE*Δ of a clinical class 1 integron (GenBank accession MN215968). **b***Luteimonas* sp. 83-4 (CP029556.1), chromosomal, not annotated in the available contig sequence. **c***P. aeruginosa* 105MG (AJ786649.2 and VLOE00000000), chromosomal, clinical class 1 integron, included in a Tn*5051*-like transposon. *Gar* variant of *P. aeruginosa* 105MG = orfPa105, previously annotated as hypothetical protein. **d***P. aeruginosa* S742_C15_BS (NFFO01000062.1), clinical class 1 integron, downstream context identical to *P. aeruginosa* 105MG, but lacking the Tn*21*-like region upstream. **e***S. enterica* subsp. *enterica* serovar *Johannesburg* FSIS1609335 (AAIUOI010000042.1), class 1 integron, downstream flanked by Tn*21* elements, IS*Pa38* and IS*110*. **f*** S. enterica* ADRDL-2811 (AAKHBQ010000151.1), downstream flanked by Tn*21* elements. **a**, **d–f** Entire contig shown. Attachment sites: *attI*, marked as blue rhomb; *attC*, marked as green rectangle; hp, hypothetical protein; *aspA*, aspartate ammonia lyase; *catB3*, chloramphenicol transferase; *aph(3′)-XV*, aminoglycoside phosphotransferase; *xerC*, tyrosine recombinase (integrase); IS*1071* (Tn*3* family): single element, including transposase and flanking inverted repeats; Tn*5051*-like *tnp* region consisting of *tnpA*: transposase, *tnpR*: resolvase and *tnpM*: putative transposition regulator; *intI1*, class 1 integron integrase; *bla*_*VIM-1*_*,* metallo-β-lactamase VIM-1; *qacE*Δ, quarternary ammonium compound resistance protein (truncated); GNAT, Gcn5-related N-acetyltransferase; *tniA–tniR* and *urf-2*, *tni* region (Tn*21*-like); *aac(6′)-Ib*, aminoglycoside acetyltransferase; *bla*_OXA-2_, class D β-lactamase OXA-2; *ant(3″)-Ia*, aminoglycoside nucleotidyltransferase; IS*Pa38* (Tn*3* family); and an IS*110* family element

We have identified *gar* in six gene cassette arrays in at least three species. These regions are within integrons and adjacent to known resistance gene cassettes, such as *aph*(*3′*)*-XV*, *bla*_OXA-2_ and *bla*_VIM-1_, providing resistance to a range of critically important antibiotics (Fig. 4). The identified genetic contexts show that *gar* has appeared as the first and only gene cassette in the integrons from PETL metagenomic samples, as the fifth in *Luteimonas* sp. 83-4, the third in *P. aeruginosa* 105MG, the fourth in *P. aeruginosa* S742_C15_BS and the first in both *S. enterica* isolates. Rearrangement of gene cassettes provides rapid adaptation in response to changing environments. The presence of *gar* in different contexts and pathogens not only shows that it could confer a selective advantage for its hosts, but that it is a gene cassette accessible to diverse microbial communities and pathogens. Integrons are present in 20% of the sequenced γ-proteobacterial genomes [45]. Class 1 integrons in particular have become very successful in pathogens and highly abundant in various human-impacted environments [46, 47]. Any compatible plasmid containing a class 1 integron could acquire the *gar* cassette and facilitate further spread. Therefore, if ecological connectivity allows, *gar* can be integrated into the genomes of diverse bacterial hosts.

So far, *gar* is a rare but already widely dispersed gene in wastewater metagenomes from at least eight countries in Europe, Asia, Africa and Australia (Table S5). Additional transfer events to other pathogens are therefore plausible. Detection of *gar* only in wastewater-impacted environments, not in metagenomes from human individuals, is coherent with its presence in a small proportion of the human population. This implies that targeted investigations of wastewater would be suitable for both initial detection and monitoring, as a single sewage sample reflects the microbiota from a very large number of individuals [48].

## Conclusions

Most clinically important resistance genes are discovered as a consequence of treatment failure or by screening clinical isolates. The combination of integron-focused functional metagenomics and in silico filtering of known resistances allowed us to explore environmental samples for already mobilised, novel resistance genes. With this approach, we discovered *gar*, a new mobile resistance gene providing high-level resistance against garosamine-containing aminoglycosides. Despite its presence in the clinics, *gar* has evaded discovery as it does not resemble previously known aminoglycoside resistance genes. The knowledge presented here offers possibilities for early surveillance, actions to reduce transmission, gene-based diagnostics and ultimately improved treatment.

## Methods

Detailed material and methods with references are found in Additional file 5.

### Bacterial strains

Metagenomic library construction and functional screenings were conducted in *E. coli* DH10β (NEB). Functional verifications were performed in *E. coli* C600Z1 (Expressys) and GAR-induced increases in aminoglycoside MICs were determined in *E. coli* BL21(DE3) (Invitrogen). *P. aeruginosa* 105MG [33] was kindly supplied by Professors C. Giske and G. Rossolini.

### Metagenomic DNA samples

DNA was extracted, pooled and amplified with three sets of primers targeting the gene cassette array of class 1 integrons from sediment samples collected from Mutha River (Pune, Maharashtra, India) and Isakavagu/Nakkavagu River (Patancheru Enviro Tech Ltd. (PETL) near Hyderabad, India) as described before [9]. In contrast to the previously performed amplification of the same samples [9], 5′ phosphorylated primers were used to generate inserts for metagenomics libraries.

### Metagenomic library preparation and functional selection

To identify mobilised novel resistance determinants, class 1 integron gene cassette libraries were prepared and screened following the protocol by Forsberg et al. [3] with some modifications. The plasmid pZE21-P_*bla*_ (pZE21-MCS1 harbouring the constitutively active promoter P_*bla*_) was linearised, dephosphorylated and ligated with the amplified class 1 integron gene cassettes to create the libraries, which were electroporated into *E. coli* DH10β.

For functional selection, 100 μl of each metagenomic library was plated on LB + Kan^50^ agar containing one of 13 different antibiotics (ciprofloxacin, trimethoprim, gentamicin, tigecycline, chloramphenicol, nitrofurantoin, rifampicin, cefotaxime, ertapenem, imipenem, meropenem, sulfamethoxazole and colistin) at three different concentrations (4 × MIC, 8 × MIC and clinical breakpoint concentration). All colonies from a single plate were scraped off, resuspended in LB + 20% glycerol and frozen at − 80 °C.

### Amplicon-PCR and sequencing

Pooled antibiotic resistant clone libraries were used as PCR templates to prepare amplicons with barcoded primers that allowed allocation of the antibiotics and concentrations used for selecting clones. Amplicons were combined into two pools (16 amplicons each), and sequencing libraries were prepared from each pool using SMRTbell™ Template Prep Kit 1.0-SPv3. The two libraries were sequenced on separate PacBio Sequel™ SMRT® cells in the Science for Life Laboratories (Uppsala, Sweden).

### Integron amplicon read analysis

Open reading frames were predicted using Prodigal [49] (v2.6.3). The predicted ORFs were searched against NCBI’s non-redundant protein database (last update 13.04.2017) and ResFinder [50] (last update 15.04.2018) using Diamond [51] (v0.9.24.125). We defined known ARGs as ORFs with identities greater than 95% and coverage greater than 85% to their homologues in the ResFinder database. Reads that contained known ARGs responsible for the respective phenotype were filtered out.

To identify promising putative novel resistance genes, a manual search of the read sets for complete ORFs that were highly abundant in their set of reads followed. Presence of the cassette amplification primers was required to ensure that the ORF originated from an integron and that it was solely responsible for the resistance phenotype.

### Functional verification of novel resistance genes

Resistance gene candidates were synthesised and tested in *E. coli* C600Z1 pZE21-MCS1 as described earlier [9]. Subsequently, *gar* was inserted into pUC19, replacing the *bla* gene and transformed into *E. coli* BL21(DE3) to test it in an expression system without any aminoglycoside resistance.

### MIC determination

Minimal inhibitory concentrations (MICs) were determined by broth microdilution in cation-adjusted Mueller-Hinton medium. Serial dilutions of tested antibiotics were prepared in triplicate in 96-well plates and inoculated with 5 × 10^5^ cells/ml per well (CLSI standard [52]) at a final volume of 200 μl. After 24 h incubation at 37 °C and 180 rpm, the optical density was measured and MIC was defined as the lowest concentration of an antimicrobial that reduced growth to OD_650_ ≤ 0.2 [53].

### Whole genome sequencing

Genomic DNA was isolated using the DNeasy® Blood & Tissue Kit (Qiagen) and sent to FIMM Technology Centre in Helsinki, Finland, for next generation sequencing. The KAPA HyperPlus Kit was used for library preparation and paired-end sequencing was performed on a MiSeq® (Illumina). The paired-end datasets were filtered and trimmed using the Trim Galore (v0.4.4) software. SPAdes [54] (v3.12.0) was used to assemble the short reads into 134 contigs with a length greater than 500 bp. The regions around the gene (Fig. 4) were scaffolded from contigs 1, 3, 62, 83 and 89 using the previously recovered integron sequence (GenBank accession: AJ786649.2) and by manually exploring De Bruijn graphs with the Bandage software [55] (v0.8.1) as well as experimental control by PCR.

### Multi-locus sequence typing

Contigs were searched against the PubMLST database [56]. *P. aeruginosa* 105MG was previously classified as ST227 [33], but whole genome sequencing resulted in a single nucleotide difference in the *mutL* allele changing the sequence type to ST235.

### Metagenome search

The abundance of *gar* was searched in 1251 public metagenomic datasets (Additional file 2). Diamond [51] (v0.9.24.125) was used to map the reads to the reference protein with 100% identity and an ORF-length greater than 20 amino acids. To study the genetic context around the novel ARG, short reads from selected metagenomic datasets were mapped to the reference sequences from *P. aeruginosa* 105MG and *Luteimonas* sp. 83-4 using Bowtie 2 [57] (v2.2.9). Integron attachment sites were detected by identifying marginal paired-end reads using the Tablet software [58] (v1.19.05).

### Phylogenetic trees

Sequences of the 1000 proteins most closely related to GAR were collected using three iterations of PSI-BLAST [59] on the NCBI non-redundant protein database. The retrieved proteins, GAR and all aminoglycoside resistance proteins from ResFinder were aligned with MAFFT [60] (v7.310). The phylogenetic tree was calculated by FastTree [61] (v2.1.9) using the maximum likelihood algorithm, Jones-Taylor-Thornton model, with 1000 bootstraps. Protein accession numbers and the full version of the tree are available in Additional files 3 and 4. The Interactive Tree Of Life (iTOL v4) online tool [62] was used to prepare the phylogenetic tree for display.

### Protein model

The I-TASSER server for protein structure and function prediction was used to create models of GAR [63]. The model with the highest confidence score (C-score, − 0.21) is shown. DeepView/Swiss-PdbViewer [64] (v4.1.0) was used to create the ribbon presentation.

## Supplementary information

**Additional file 1.** Supplementary Material. **Table S1.** Cation-dependency: MIC gentamicin [μg/ml] of GAR in different media. **Table S2.** Chemical structure of the tested aminoglycosides and resistance profile conveyed by GAR. **Table S3.** Predicted and determined resistances of *Pseudomonas aeruginosa* 105MG. **Table S4.** Predicted resistances of *Luteimonas* sp. 83–4. **Table S5.** Occurrence of *gar* in 1251 metagenomic datasets.

**Additional file 2. **List of metagenomes searched for the presence of *gar*.

**Additional file 3.** Accession numbers of protein sequences used to calculate the phylogenetic tree.

**Additional file 4.** Phylogenetic tree of AMEs, GAR and the most closely related proteins in Newick format.

**Additional file 5.** Detailed material and methods.

## Data Availability

Functional metagenomics sequencing data are available in Bioproject PRJNA555822, *P. aeruginosa* 105MG assembly in GenBank, accession VLOE00000000; and genetic context of *gar* from functional metagenomics in GenBank, accession MN215968.

## Abbreviations

AAC: Aminoglycoside N-acetyltransferase
AME: Aminoglycoside modifying enzyme
ANT: Aminoglycoside O-nucleotidyltransferase
APH: Aminoglycoside O-phosphotransferase
ARG: Antibiotic resistance gene
ICE: Integrative conjugative element
MIC: Minimal inhibitory concentration
MLST: Multi-locus sequence typing
ORF: Open reading frame
ST: Sequence type
