# Supplementary material for: Discovery of a novel integron-borne aminoglycoside resistance gene present in clinical pathogens by screening environmental bacterial communities
Source: Microbiome. 2020 Mar 20;8:41. doi: 10.1186/s40168-020-00814-z (PMC7085159; doi:10.1186/s40168-020-00814-z)
Supplement: Supplementary file 1 — Additional file 1. Supplementary Material. Table S1. Cation-dependency: MIC gentamicin [μg/ml] of GAR in different media. Table S2. Chemical structure of the tested aminoglycosides and resistance profile conveyed by GAR. Table S3. Predicted and determined resistances of Pseudomonas aeruginosa 105MG. Table S4. Predicted resistances of Luteimonas sp. 83–4. Table S5. Occurrence of gar in 1251 metagenomic datasets. [file 40168_2020_814_MOESM1_ESM.pdf]

**Supplementary Material**

Table S1: Cation-dependency: MIC gentamicin [µg/ml] of GAR in different media.
Strain: *E. coli* C600Z1, induction: 250 ng/ml anhydrotetracycline. Median of two – four biological replicates.

| | **Induced** | **Increase of MIC** | **Not induced** | **Increase of MIC** |
|---|---|---|---|---|
| **LB plates, E-test** | | | | |
| pZE21-*gar* | 128 | **171** | 0.625 | **2** |
| pZE21-MCS1 | 0.75 | | 0.345 | |
| **MH plates, E-test** | | | | |
| pZE21-*gar* | 64 | **1000** | 0.157 | **2** |
| pZE21-MCS1 | 0.064 | | 0.064 | |
| **LB broth, BMD** | | | | |
| pZE21-*gar* | 5.76 | **3** | 4.32 | **2** |
| pZE21-MCS1 | 2.16 | | 2.16 | |
| **MH broth, BMD** | | | | |
| pZE21-*gar* | 184 | **657** | 0.4 | **2** |
| pZE21-MCS1 | 0.28 | | 0.2 | |
| **Cation-adjusted MH broth, BMD** | | | | |
| pZE21-*gar* | 121 | **2017** | 0.1 | **2** |
| pZE21-MCS1 | 0.06 | | 0.05 | |

In order to further confirm and quantify the level of gentamicin resistance conferred by GAR, we determined MIC values by standard broth microdilution in LB medium (Table S1). Here, MIC values were raised only by factor 3. It was shown earlier that divalent cations increase aminoglycoside MIC values [1, 2]. Consequently, the addition of agar in the first test, containing an unknown amount of cations, resulted in increased resistance. A hypothesis behind this phenomenon is that mono- and divalent cations, especially $Mg^{2+}$ and $Ca^{2+}$, antagonize the antibiotic effect most likely by displacement of the aminoglycoside from a site at the cell wall thereby stopping uptake, as shown for both Gram-negative and Gram-positive bacteria [3, 4]. We then performed broth microdilution (BMD) in MH broth and E-test on MH plates, which both led to a more than 600x increase of the MIC. The highest resistance level was measured in the medium with the highest cation concentration, cation-adjusted MH broth (20-25 mg $Ca^{2+}$/L and 10-12.5 mg $Mg^{2+}$/L, equalling about 500 µM of each) [5]. However, this increase in MIC could not be found in the negative controls without resistance gene suggesting that this is not the complete explanation. The observation that the MIC was only changed in a cation-dependent fashion for strains expressing *gar* indicates a more direct interaction. GAR contains an AAA domain and shares similarities with nucleotide and gluconate kinases (Fig. 2, main article). These, the known aminoglycoside nucleotidyltransferases and aminoglycoside phosphotransferases as well as other P-loop containing NTPases are $Mg^{2+}$-dependent enzymes [6-10]. Ideally, the concentration of free $Mg^{2+}$ in the cytosol is kept stable between 1 – 5 mM and, additionally, a large amount of $Mg^{2+}$ is bound and required by the ribosomes [11]. Intracellular free $Mg^{2+}$ can vary between 1 µM and 10 mM depending on the extracellular concentration [12]. The concentration of divalent cations in cation-adjusted MH medium is sufficient for all cellular functions but requires active enrichment of $Mg^{2+}$ in the cells. We cannot exclude that extremely low concentrations in the medium might cause lower levels of free $Mg^{2+}$ in the cytosol and lead to the observed decrease in resistance by limiting GAR activity, although this remains to be shown.

Since cation concentrations influenced MIC values profoundly, we decided to perform all subsequent experiments in cation-adjusted MH broth. In order to reliably compare GAR activity against a broad palette of aminoglycosides, we additionally chose to change vector and bacterial strain. The vector pZE21 includes an APH(3') kanamycin resistance (although not active against gentamicin) and thus all mentioned MIC determinations were performed in presence of 50 µg/ml kanamycin. The strain *E. coli* C600Z1 also harbours a chromosomal spectinomycin resistance determinant, making it less suitable. We therefore cloned the ORF *gar* into pUC19 replacing the ampicillin resistance gene. The plasmid pUC19-*gar* was then transformed into *E. coli* BL21(DE3), which contains no internal resistance gene and requires no induction of *gar* expression. The resulting strain showed a 2760x gentamicin MIC increase in cation-adjusted MH (Fig. 1, main article).

Table S2: Chemical structure of the tested aminoglycosides and resistance profile conveyed by GAR. Suspected target garosamine is marked. Chemical structures were prepared using ACD/ChemSketch (Freeware) 2018.1.1.

| **Antibiotic** | **Structure** | **Manufacturer and catalog number** | **MIC increase (fold-change)** |
|---|---|---|---|
| Netilmicin | 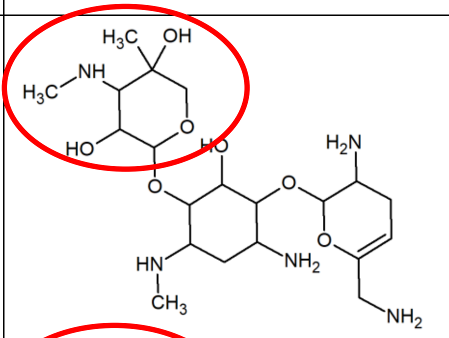 | Sigma 1460500 USP | 5140 |
| G418 | 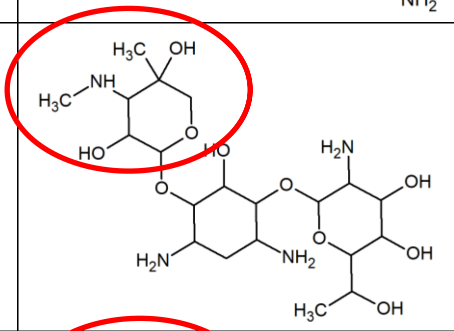 | Sigma G1279 | 4850 |
| Isepamicin | 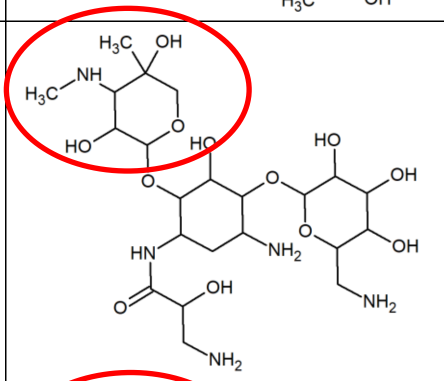 | TOKU-E I042 | 4110 |
| Sisomicin | 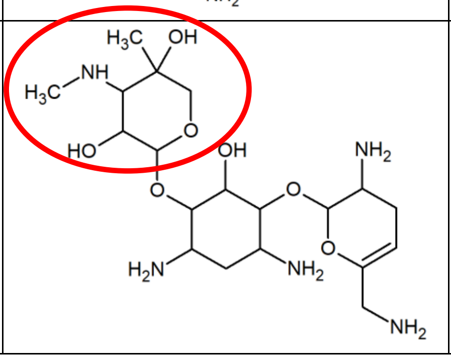 | Sigma 1612801 USP | 3430 |

| **Antibiotic** | **Structure** | **Manufacturer and catalog number** | **MIC increase (fold-change)** |
|---|---|---|---|
| Micronomicin | 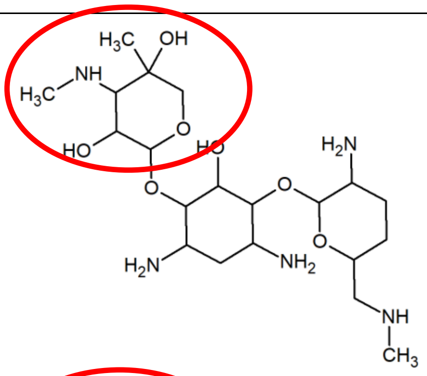 | TOKU-E M018 | 3080 |
| Gentamicin | 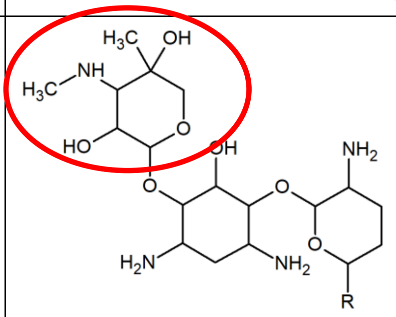<br>R = C1 – $CH_2NH_2$, C2 – $C_2NH_6$, or C3 – $C_3NH_8$ | Sigma G3632 | 2760 |
| Tobramycin | 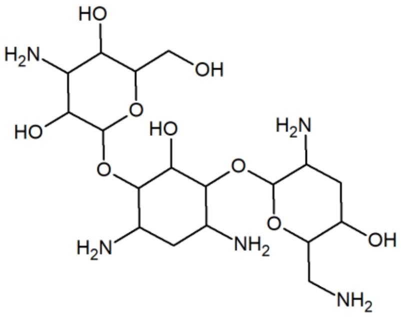 | Sigma T4014 | 16 |
| Kanamycin | 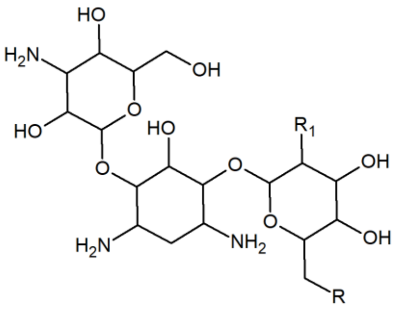<br>R/$R_1$ = $NH_2$ or OH | Sigma K4000 | 8 |

| Antibiotic | Structure | Manufacturer and catalog number | MIC increase (fold-change) |
|---|---|---|---|
| Amikacin | 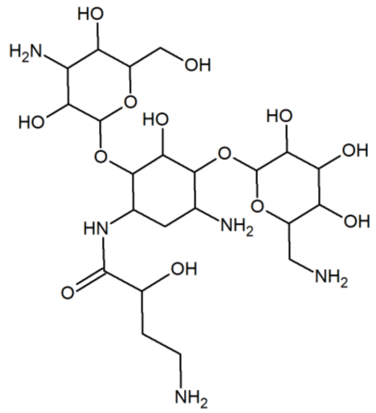 | Sigma A3650 | 8 |
| Paromomycin | 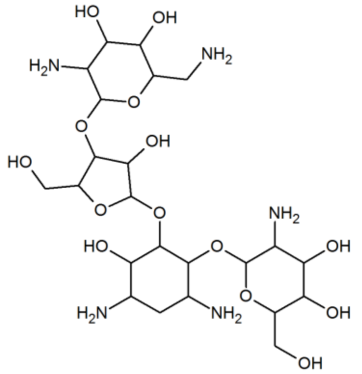 | Sigma P5057 | 8 |
| Ribostamycin | 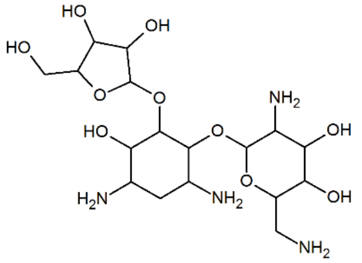 | Sigma R2255 | 4 |
| Apramycin | 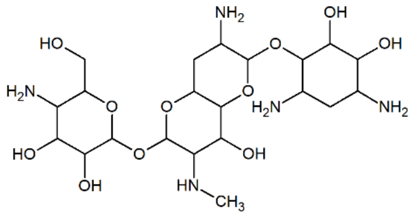 | Sigma A2024 | 4 |

| **Antibiotic** | **Structure** | **Manufacturer and catalog number** | **MIC increase (fold-change)** |
|---|---|---|---|
| Hygromycin B | 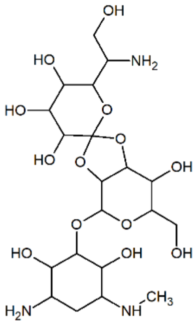 | Sigma H3274 | 3 |
| Streptomycin | 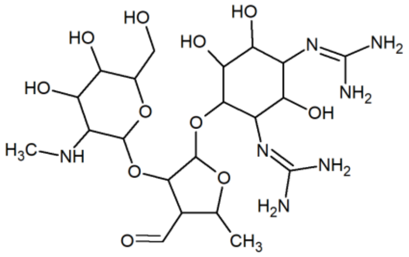 | Sigma S6501 | 2 |
| Spectinomycin | 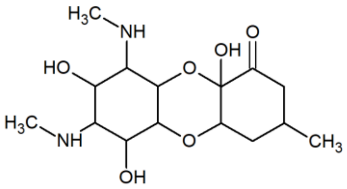 | Sigma S4014 | 1 |

Table S3: Predicted and determined resistances of ***Pseudomonas aeruginosa* 105MG.**

This isolate (sequenced in this study) contains five aminoglycoside resistance genes including *gar*. It is also resistant against cabapenems, cephalosporins, penicillins and fluoroquinolones [13] and harbours resistance genes against chloramphenicol, fosfomycin, sulfonamides, trimethoprim and tetracycline.

| | *aph(3)-IIb* | *aac(6)-31* | *aadA6* | *aac(6)-Il* | *gar* | Determined resistance |
|---|---|---|---|---|---|---|
| Kanamycin | X | X | - | X | - | X |
| Gentamicin | - | X | - | X | X | X |
| Netilmicin | - | X | - | X | X | X |
| Sisomicin | - | X | - | X | X | X |
| G418 | X | - | - | - | X | X |
| Micronomicin | - | - | - | - | X | X |
| Isepamicin | X | X | - | X | X | X |
| Amikacin | X | X | - | X | - | X |
| Tobramycin | - | X | - | X | - | X |
| Paromomycin | X | - | - | - | - | X |
| Ribostamycin | X | X | - | X | - | X |
| Hygromycin B | ? | ? | - | ? | - | X |
| Apramycin | ? | ? | - | ? | - | - |
| Spectinomycin | ? | - | X | - | - | X |
| Streptomycin | ? | - | X | - | - | X |

Resistance profiles of the listed aminoglycoside modifying enzymes: [14] and references therein.

Table S4: Predicted resistances of ***Luteimonas* sp. 83-4.**

This isolate (complete genome, accession CP029556.1) contains two aminoglycoside resistance genes including *gar*.

| | *aph(3')-XV* | *gar* |
|---|---|---|
| Kanamycin | X | - |
| Gentamicin | - | X |
| Netilmicin | - | X |
| Sisomicin | - | X |
| G418 | X | X |
| Micronomicin | - | X |
| Isepamicin | X | X |
| Amikacin | X | - |
| Tobramycin | - | - |
| Paromomycin | X | - |
| Ribostamycin | X | - |
| Hygromycin B | ? | - |
| Apramycin | ? | - |
| Spectinomycin | ? | - |
| Streptomycin | X | - |

Table S5: Occurrence of *gar* in 1251 metagenomic datasets.

The number of paired-end reads mapping to *gar* with 100 % identity over the length of at least 20 amino acids are shown. Reads from selected metagenomic datasets (ERR1713392, ERR1713378) were mapped to DNA sequences containing *gar* and surrounding attachment sites. Paired-end reads covered *gar* and the adjacent attachment site unambiguously, confirming that *gar* is present as a gene cassette in both metagenomes.

| Metagenome | Raw count | Environment | Country | Latitude | Longitude |
|---|---|---|---|---|---|
| ERR1713392 | 15 | Wastewater/sludge | Sweden | 57.7089 | 11.9746 |
| ERR1713378 | 13 | Wastewater/sludge | Nigeria | 7.3775 | 3.9470 |
| ERR1726005 | 7 | Wastewater/sludge | Sweden | 57.7089 | 11.9746 |
| ERR1726006 | 6 | Wastewater/sludge | Sweden | 57.7089 | 11.9746 |
| ERR1725942 | 5 | Wastewater/sludge | Australia | -37.9145 | 144.6432 |
| ERR1414237 | 4 | Wastewater/sludge | Sweden | 59.3107 | 18.1084 |
| ERR1725996 | 4 | Wastewater/sludge | Nigeria | 7.3775 | 3.9470 |
| ERR1713407 | 3 | Wastewater/sludge | Viet Nam | 10.8231 | 106.6297 |
| 104 | 2 | Sediment | India | 18.5104 | 73.8399 |
| ERR2592328 | 2 | Wastewater/sludge | Australia | -35.3452 | 149.0950 |
| ERR1713369 | 2 | Wastewater/sludge | Kenya | 1.2921 | 36.8219 |
| ERR1414243 | 1 | Wastewater/sludge | Sweden | 59.3556 | 18.2271 |
| ERR1414272 | 1 | Wastewater/sludge | Sweden | 59.3556 | 18.2271 |
| 111 | 1 | Wastewater/sludge | India | 19.0760 | 72.8777 |
| ERR1713332 | 1 | Wastewater/sludge | Australia | -35.3452 | 149.0950 |
| ERR1713333 | 1 | Wastewater/sludge | Australia | -37.9145 | 144.6432 |
| ERR1713372 | 1 | Wastewater/sludge | Luxembourg | 49.5096 | 5.9221 |

Table S6: Composition of the 1251 metagenomics datasets.

See Additional file 2 for the extensive list.

| Source | Nr. of metagenomes |
|---|---|
| Wastewater/sludge | 453 |
| Human, individuals | 339 |
| Marine environments | 203 |
| Hydrothermal vent | 143 |
| River/lake sediment | 46 |
| Estuary | 19 |
| River/lake sediment, pharmaceutical impact | 16 |
| Soil | 16 |
| Thermophilic anaerobic methanogenic reactor | 5 |
| Cold seep sediment | 3 |
| Alkaline water | 3 |
| Oyster gut | 2 |
| Crater lake (stromatolite) | 2 |
| Hot spring | 1 |

## References


1. Garrod LP, Waterworth PM. Effect of medium composition on the apparent sensitivity of Pseudomonas aeruginosa to gentamicin. J Clin Pathol. 1969;22:534-8. doi: 10.1136/jcp.22.5.534.
2. D'Amato RF, Thornsberry C, Baker CN, Kirven LA. Effect of calcium and magnesium ions on the susceptibility of Pseudomonas species to tetracycline, gentamicin polymyxin B, and carbenicillin. Antimicrob Agents Chemother. 1975;7:596-600. doi: 10.1128/aac.7.5.596.
3. Bryan LE, Van Den Elzen HM. Effects of membrane-energy mutations and cations on streptomycin and gentamicin accumulation by bacteria: a model for entry of streptomycin and gentamicin in susceptible and resistant bacteria. Antimicrob Agents Chemother. 1977;12:163-77. doi: 10.1128/aac.12.2.163.
4. Beggs WH, Andrews FA. Inhibition of dihydrostreptomycin binding to Mycobacterium smegmatis by monovalent and divalent cation salts. Antimicrob Agents Chemother. 1976;9:393-6. doi: 10.1128/aac.9.3.393.
5. CLSI. Methods for dilution antimicrobial susceptibility tests for bacteria that grow aerobically: M07-A9; approved standard. 9. ed. Wayne, PA 19087 USA: Committee for Clinical Laboratory Standards Institute; 2012.
6. Das U, Wang LK, Smith P, Munir A, Shuman S. Structures of bacterial polynucleotide kinase in a michaelis complex with nucleoside triphosphate (NTP)-Mg2+ and 5'-OH RNA and a mixed substrate-product complex with NTP-Mg2+ and a 5'-phosphorylated oligonucleotide. J Bacteriol. 2014;196:4285-92. doi: 10.1128/JB.02197-14.
7. Kraft L, Sprenger GA, Lindqvist Y. Conformational changes during the catalytic cycle of gluconate kinase as revealed by X-ray crystallography. J Mol Biol. 2002;318:1057-69. doi: 10.1016/S0022-2836(02)00215-2.
8. Cox G, Stogios PJ, Savchenko A, Wright GD. Structural and molecular basis for resistance to aminoglycoside antibiotics by the adenylyltransferase ANT(2”)-Ia. MBio. 2015;6. doi: 10.1128/mBio.02180-14.
9. Labby KJ, Garneau-Tsodikova S. Strategies to overcome the action of aminoglycoside-modifying enzymes for treating resistant bacterial infections. Future Med Chem. 2013;5:1285-309. doi: 10.4155/fmc.13.80.
10. Miller JM, Enemark EJ. Fundamental Characteristics of AAA+ Protein Family Structure and Function. Archaea. 2016;2016:9294307. doi: 10.1155/2016/9294307.
11. Nierhaus KH. Mg2+, K+, and the ribosome. J Bacteriol. 2014;196:3817-9. doi: 10.1128/JB.02297-14.
12. Hurwitz C, Rosano CL. The intracellular concentration of bound and unbound magnesium ions in *Escherichia coli*. J Biol Chem. 1967;242:3719-22. http://www.ncbi.nlm.nih.gov/pubmed/5341484.
13. Giske CG, Libisch B, Colinon C, Scoulica E, Pagani L, Füzi M, et al. Establishing clonal relationships between VIM-1-like metallo-beta-lactamase-producing Pseudomonas aeruginosa strains from four European countries by multilocus sequence typing. J Clin Microbiol. 2006;44:4309-15. doi: 10.1128/JCM.00817-06.
14. Ramirez MS, Tolmasky ME. Aminoglycoside modifying enzymes. Drug Resist Updat. 2010;13:151-71. doi: 10.1016/j.drup.2010.08.003.
