# Supplementary material for: Discovery of a novel integron-borne aminoglycoside resistance gene present in clinical pathogens by screening environmental bacterial communities
Source: Microbiome. 2020 Mar 20;8:41. doi: 10.1186/s40168-020-00814-z (PMC7085159; doi:10.1186/s40168-020-00814-z)
Supplement: Supplementary file 5 — Additional file 5. Detailed material and methods. [file 40168_2020_814_MOESM5_ESM.pdf]

Detailed Material and Methods

Bacterial strains

Metagenomic library construction and functional screenings were conducted in *E. coli* DH10β (NEB). Functional verifications were performed in *E. coli* C600Z1 (Expressys, Germany) and GAR-induced increases in aminoglycoside MICs were determined in *E. coli* BL21(DE3) (Invitrogen, Thermo Fisher Scientific, USA). *P. aeruginosa* 105MG [1] was kindly supplied by Professors C. Giske and G. Rossolini.

Metagenomic DNA samples

DNA was extracted, pooled and amplified with three sets of primers targeting the gene cassette array of class 1 integrons from sediment samples collected at Mutha River (Pune, Maharashtra, India) and Isakavagu/Nakkavagu River (Patancheru Enviro Tech Ltd. (PETL) near Hyderabad, India) as described before [2]. In contrast to the previously performed amplification of the same samples [2], 5' phosphorylated primers were used to generate inserts for metagenomics libraries.

Metagenomic library preparation and functional selection

To identify mobilized novel resistance determinants, class 1 integron gene cassette libraries were prepared and screened following the protocol by Forsberg *et al.*, 2014 [3] with some modifications. The vector pZE21-MCS1 (Expressys, Germany) was modified for library preparation by insertion of the constitutively active promoter P_{bla} and its ribosomal binding site via the restriction sites KpnI and HincII. The resulting plasmid pZE21-P_{bla} was linearized (Phusion High-Fidelity DNA Polymerase (Thermo Fisher Scientific, USA)) with the primers pZE21-for and pZE21-Pbla-rev (Table S6) and subsequently dephosphorylated using FastAP (Thermo Fisher Scientific, USA).

Table S6: Primers used to linearize pZE21-P_{bla}.

**Primer**	**Sequence 5' → 3'**	**Reference**
pZE21-for	GACGGTATCGATAAGCTTGAT	[3]
pZE21-Pbla-rev	GACTCTTCCTTTTTCAATATTATTGAA	This study

Libraries of class 1 integron gene cassettes were created by overnight-ligation (Fast Link ligation kit (Epicentre, Lucigen, USA)) of linearized pZE21-P_{bla} with gene cassettes amplified from Pune and PETL metagenomic DNA samples at a molar ratio of 1:5 followed by electroporation into *E. coli* DH10β. After recovery 1 µl, 0.1 µl and 0.01 µl of each library was plated on LB agar containing 50 µg/ml kanamycin to determine library size, plate count and average insert size. The rest of the libraries was inoculated into 10 ml LB + Kan^{50} and incubated for 18 h at 37 °C, 180 rpm. Libraries were aliquoted and frozen in 20 % glycerol at -80 °C. Library sizes were estimated as multiplication of the average insert size by the number of colony forming units after transformation recovery. Insert size distribution was estimated by PCR amplification and gel electrophoresis of inserts from 10 randomly picked clones of each library.
For functional selection 100 µl of each metagenomic library were plated on LB + Kan^{50} agar containing additionally one of 13 antibiotics at 4x, 8x minimal inhibitory concentration (MIC) and/or clinical breakpoint concentration for *E. coli* DH10β. Plates were incubated at 37 °C for 24 h. No resistance against tigecycline or nitrofurantoin was found (Table S7). The other screening combinations resulted in at least 15 to up to several thousand colonies on each plate.

Table S7: Functional screening of gene cassette libraries against different antibiotics.
Clinical breakpoints according to EUCAST breakpoint tables v_8.1 (2018). For functional selection 100 µl of each metagenomic library were plated on LB + Kan^{50} agar containing additionally one of 13 antibiotics at 4x, 8x minimal inhibitory concentration and/or clinical breakpoint concentration for *E. coli* DH10β. Plates were incubated at 37 °C for 24 h.

**Antibiotic**	**4x MIC [µg/ml]**	**8x MIC [µg/ml]**	**Clinical breakpoint [µg/ml]**
Ciprofloxacin	**0.012**	**0.024**	*0.5*
Trimethoprim	0.8	1.6	**4**
Gentamicin*	4	**8**	4 → n.t.
Tigecycline	*0.8*	*1.6*	*2*
Imipenem	**0.8**	**1.6**	8 → n.t.
Chloramphenicol	4	**8**	8 → n.t.
Nitrofurantoin	*4*	*8*	*64*
Rifampicin	32	**64**	- → n.t.
Ertapenem	0.016	**0.032**	**1**
Meropenem	**0.1**	**0.2**	*8*
Colistin**	**1**	**2**	2 → n.t.
Sulfamethoxazole***	64	**128**	- → n.t.
Cefotaxime	0.2	**0.4**	**2**

* based on the MIC of *E. coli* DH10β pZE21-P_{bla} containing a kanamycin resistance gene on LB + Kan^{50}.

** colistin cannot be reliably used in agar plates due to insufficient diffusion in solid media and adherence to polystyrene [4, 5], liquid cultures were inoculated with 100 µl of each metagenomic library in glass tubes.

*** tested on MH agar, containing no folate.

Highlighted in grey: no growth, highlighted in bold: sequenced, n.t. = not tested.

In many functional metagenomics studies single clones were picked and sequenced [6, 7], which significantly limits the sensitivity of the method. Since we expected potential novel resistance factors to be dominated by known ones, we chose instead to sequence all resistant clones [3]. All colonies from a single plate were scraped off using a sterile disposable cell scraper (Sarstedt, Germany) and resuspended in 500 µl LB + 20 % glycerol. Colonies resulting from the same antibiotic and metagenomic DNA sample (Pune or PETL) were combined before they were frozen at -80 °C. Thus, the functionally selected libraries contained a mixture of integron gene cassettes by using all three primer pairs.

Amplicon-PCR and sequencing

The selected and pooled antibiotic resistant clone libraries were thawed and 300 µl were washed twice in PBS buffer. Cells were subsequently pelleted a third time in nuclease-free H_2O, re-suspended in 50 µl H_2O and used as PCR template. For plates with 1-100 colonies, 1-2 µl of plate scrape lysate was used; for plates with 100-2000 colonies, 0.1 µl was used; and for plates with >2000 colonies, 0.01 µl was used as template. Amplicons were prepared with Phusion High-Fidelity DNA Polymerase and primers binding directly up- and downstream of the insertion site within pZE21-P_{bla} (Table S8). The amplicon PCR primers contain sample specific barcodes that allow allocation to the antibiotic and concentration used for selecting clones.

Table S8: Primers used to prepare amplicons for sequencing.

The plasmid-binding region is marked in red and the first 16 barcodes from the list recommended for SMRT sequencing were used. See: https://github.com/PacificBiosciences/Bioinformatics-Training/blob/master/barcoding/pacbio_384_barcodes.fasta

Forward barcoded primer 5' → 3'	
**F1**	TCAGACGATGCGTCATGAATTCATTAAAGAGGAGAAAGGTAC
**F2**	CTATACATGACTCTGCGAATTCATTAAAGAGGAGAAAGGTAC
**F3**	TACTAGAGTAGCACTCGAATTCATTAAAGAGGAGAAAGGTAC
**F4**	TGTGTATCAGTACATGGAATTCATTAAAGAGGAGAAAGGTAC
**F5**	ACACGCATGACACACTGAATTCATTAAAGAGGAGAAAGGTAC
**F6**	GATCTCTACTATATGCGAATTCATTAAAGAGGAGAAAGGTAC
**F7**	ACAGTCTATACTGCTGGAATTCATTAAAGAGGAGAAAGGTAC
**F8**	ATGATGTGCTACATCTGAATTCATTAAAGAGGAGAAAGGTAC
**F9**	CTGCGTGCTCTACGACGAATTCATTAAAGAGGAGAAAGGTAC
**F10**	GCGCGATACGATGACTGAATTCATTAAAGAGGAGAAAGGTAC
**F11**	CGCGCTCAGCTGATCGGAATTCATTAAAGAGGAGAAAGGTAC
**F12**	GCGCACGCACTACAGAGAATTCATTAAAGAGGAGAAAGGTAC
**F13**	ACACTGACGTCGCGACGAATTCATTAAAGAGGAGAAAGGTAC
**F14**	CGTCTATATACGTATAGAATTCATTAAAGAGGAGAAAGGTAC
**F15**	ATAGAGACTCAGAGCTGAATTCATTAAAGAGGAGAAAGGTAC
**F16**	TAGATGCGAGAGTAGAGAATTCATTAAAGAGGAGAAAGGTAC

Reverse barcoded primer 5' → 3'	
**R1**	ATGACGCATCGTCTGATCGATATCAAGCTTATCGATACC
**R2**	GCAGAGTCATGTATAGTCGATATCAAGCTTATCGATACC
**R3**	GAGTGCTACTCTAGTATCGATATCAAGCTTATCGATACC
**R4**	CATGTACTGATACACATCGATATCAAGCTTATCGATACC
**R5**	AGTGTGTCATGCGTGTTCGATATCAAGCTTATCGATACC
**R6**	GCATATAGTAGAGATCTCGATATCAAGCTTATCGATACC
**R7**	CAGCAGTATAGACTGTTCGATATCAAGCTTATCGATACC
**R8**	AGATGTAGCACATCATTCGATATCAAGCTTATCGATACC
**R9**	GTCGTAGAGCACGCAGTCGATATCAAGCTTATCGATACC
**R10**	AGTCATCGTATCGCGCTCGATATCAAGCTTATCGATACC
**R11**	CGATCAGCTGAGCGCGTCGATATCAAGCTTATCGATACC
**R12**	TCTGTAGTGCGTGCGCTCGATATCAAGCTTATCGATACC
**R13**	GTCGCGACGTCAGTGTTCGATATCAAGCTTATCGATACC
**R14**	TATACGTATATAGACGTCGATATCAAGCTTATCGATACC
**R15**	AGCTCTGAGTCTCTATTCGATATCAAGCTTATCGATACC
**R16**	TCTACTCTCGCATCTATCGATATCAAGCTTATCGATACC

The resulting 32 amplicon PCR products were purified (PCR purification kit, Qiagen, Germany), quantified using Qubit® Fluorometer and quality was assured by Nanodrop™ spectrophotometer. The amplicons were combined into two pools (16 amplicons each, Table S9). Sequencing libraries were prepared from each pool using the SMRTbell™ Template Prep Kit 1.0-SPv3 and the two libraries were sequenced on separate PacBio Sequel™ SMRT® cells in the Science for Life Laboratories (Uppsala, Sweden).

Table S9: Contents of the pooled selection libraries.

Pool 1 Barcode	Pool 1 Sample	Pool 2 Barcode	Pool 2 Sample
1	Cip 4x Pune	1	Rif 8x Pune
2	Cip 4x PETL	2	Rif 8x PETL
3	Cip 8x PETL	3	Mer 4x Pune
4	Tri clB Pune	4	Mer 4x PETL
5	Tri clB PETL	5	Mer 8x Pune
6	Imi 4x Pune	6	Mer 8x PETL
7	Imi 4x PETL	7	Sul 8x Pune
8	Imi 8x PETL	8	Sul 8x PETL
9	Gen 8x Pune	9	CTX 8x Pune
10	Gen 8x PETL	10	CTX 8x PETL
11	Cam 8x Pune	11	CTX clB Pune
12	Cam 8x PETL	12	CTX clB PETL
13	Ert 8x Pune	13	Col 4x Pune
14	Ert 8x PETL	14	Col 4x PETL
15	Ert clB Pune	15	Col 8x Pune
16	Ert clB PETL	16	Col 8x PETL

Integron amplicon read analysis

Sequencing of amplicons resulted in 419709 reads. Based on the retrieved barcodes, 382332 could be assigned to the antibiotic and concentration used to select resistant clones. Open reading frames were predicted using Prodigal [8] (v2.6.3). The predicted ORFs were searched against NCBI's non-redundant protein database (last update 13.04.2017) and ResFinder [9] (last update 15.04.2018) using Diamond [10] (v0.9.24.125). We defined known ARGs as the ORFs with identity greater than 95 % and coverage greater than 85 % to their homologs in the ResFinder database. Reads that contain known ARGs which are responsible for the respective phenotype were filtered out. The remaining 147151 reads consisted of 46403 unique reads (11 % of all reads) with an average length of 1540 bp and they include 48562 unique predicted ORFs. Each of the 32 antibiotic selection amplicons thus resulted in an average of 1450 reads with no known antibiotic resistance gene.

To identify promising putative novel resistance genes, a manual search using five criteria followed: An ORF needed to be (i) complete and (ii) highly abundant in its set of reads while (iii) not common in the other selection sets. To ensure that the ORF originated from an integron (iv) both binding sites for the primers used to amplify the gene cassettes should be present in the read. Furthermore, (v) the candidate ORF should be the only ORF present that could explain the resistance phenotype. Using this approach, candidate ORFs with little or no resemblance to any known resistance factor were chosen for functional verification. Most noticeable was an ORF (designated *gar* = **g**arosamine-specific **a**minoglycoside **r**esistance) which occurred 1692 times in the gentamicin selection set and scarcely in the rest of the functional selection sets (Table 1, main article).

EMBOSS pepstats (https://www.ebi.ac.uk/Tools/seqstats/emboss_pepstats/) was used to estimate molecular weight and isoelectric point (pI) of GAR.

Functional verification of novel resistance genes

Resistance gene candidates were synthesized and subcloned into pZE21-MCS1 using KpnI and BamHI restriction sites by GeneArt Gene Synthesis (Thermo Fisher Scientific, Germany) as described earlier [2]. The recombinant plasmids were electroporated into *E. coli* C600Z1 and MICs were determined using E-test stripes on agar plates or by broth microdilution with the addition of 250 ng/ml anhydrotetracycline as inducer of the $P_{LtetO-1}$ promoter to ensure maximal expression. The same strain with empty vector was used as negative control. Resistance activity of the ORF designated *gar* could be verified on gentamicin.

Construction of pUC19-*gar*

An expression system without any aminoglycoside resistance except *gar* was created. *Gar* was amplified from pZE21-*gar* using the primers pZE21-SpeI and pZE21-R and inserted into pUC19, replacing the *bla* gene. The vector pUC19 was linearized with the primers pUC-NcoI and pUC-SpeI (Table S10). After digest with NcoI and SpeI, fragments were ligated and transformed into *E. coli* BL21(DE3).

Table S10: Primers used to construct pUC19-*gar*.

**Primer**	**Sequence 5' → 3'**
pZE21-SpeI	GACGCACTAGTCGAATTCATTAAAGAG
pZE21-R	CTCTAGCACGCGTACCATGG
pUC-NcoI	GTCAGACCATGGTTACTCATATATAC
pUC-SpeI	CAATATTACTAGTGCATTTATCAGGG

MIC determination

Minimal inhibitory concentrations (MICs) were determined by broth microdilution (BMD) in cation-adjusted MH medium. Serial dilutions of the tested antibiotics were prepared in triplicates in 96-well plates and inoculated with $5*10^5$ cells/ml [11] in each well at a final volume of 200 µl. After 24 h incubation at 37 °C and 180 rpm optical density was measured (Spectramax 340PC 384, Molecular Devices, USA) and the MIC was defined as the lowest concentration of an antimicrobial that reduced growth to an $OD_{650} \leq 0.2$ [12]. Most antibiotics were bought as sulphate salts, the presented MIC values refer to the active base concentration of each antibiotic. Median values of at least three independent biological replicates are shown.

Whole genome sequencing (WGS)

Genomic DNA was isolated using the DNeasy® Blood & Tissue Kit (Qiagen, Germany) and sent to FIMM Technology Centre in Helsinki, Finland, for next generation sequencing. The KAPA HyperPlus Kit (Roche, Switzerland) was used for library preparation with a fragment size of ~600 bp and paired-end sequencing (2x 300 bp) was performed on an Illumina MiSeq® with v3 chemistry.

The paired-end datasets were filtered and trimmed using Trim Galore software (v0.4.4; --retain_unpaired --paired -q 20 --length 20) (https://www.bioinformatics.babraham.ac.uk/projects/trim_galore/). SPAdes [13] (v3.12.0; --careful -k 21, 33, 55, 77, 99, 127) was used to assemble the short reads into 134 contigs with a length greater than 500 bp. Scaffolding the regions around the gene was preformed using the previously recovered Integron sequence (GenBank accession: AJ786649.2) and by manually exploring De Bruijn graphs with the Bandage software [14] (v0.8.1) as well as experimental control by PCR (Table S11). PCR and Sanger sequencing allowed assembly of the contigs 1, 3, 62, 83 and 89 based on the sequence and synteny of AJ786649.2 resulting in the context shown in Figure 4C (main article).

Table S11: Primer pairs used to verify the assembly of the *P. aeruginosa* 105MG integron containing *gar*.

**Primer**	**Sequence 5' → 3'**
gar_out	CGTTGCTTGGACTTCATTAG
Sul1_in	CGGGGCTCAAGAAAAATCC
Sul1_out	ACCTTCGACCCGAAGAC
GNAT_in	CGCTCGTATAGGCCAC
GNAT_out	CTCCGCGCTGATCGAG
trans_in	GGCTGGAAGCCCTTTATG

Multi-locus sequence typing (MLST)

Contigs were searched against the MLST scheme for *P. aeruginosa* deposited in the PubMLST database [15] (https://pubmlst.org/paeruginosa/) using the BLASTN algorithm. *P. aeruginosa* 105MG resulted in a perfect match with 100 % identity and 100 % coverage to ST235 (*acsA*: 38, *aroE*: 11, *guaA*: 3, *mutL*: 13, *nuoD*: 1, *ppsA*: 2, *trpE*: 4). *P. aeruginosa* 105MG was previously assorted to ST227 (*acsA*: 38, *aroE*: 11, *guaA*: 3, *mutL*: 9, *nuoD*: 1, *ppsA*: 2, *trpE*: 4) [1]. ST227 defers from ST235 by one mismatch at position nine in the gene *mutL*. Mapping of the WGS raw paired-end reads using Bowtie 2 [16] (v2.2.9) resulted in a single nucleotide difference in the *mutL* allele with no variation at position nine (coverage 27x), which changed the sequence type to ST235. *P. aeruginosa* S742_C15_BS resulted in a perfect match to ST111 (*acsA*: 17, *aroE*: 5, *guaA*: 5, *mutL*: 4, *nuoD*: 4, *ppsA*: 4, *trpE*: 3).

Conjugation

Equal volumes of donor and recipient bacteria were mixed on a LB agar plate and incubated at 37 °C or 30 °C overnight. Bacteria were scratched off the plate, resuspended in physiological NaCl solution and plated on selective agar plates in appropriate dilutions. Conjugation between *P. aeruginosa* 105MG and *E. coli* C600Z1 was performed at 37 °C and plated on ECC agar containing 50 µg/ml gentamicin. Conjugation between *P. aeruginosa* 105MG and *P. putida* KT2442 GFP was performed at 30 °C and plated on LB agar containing 50 µg/ml gentamicin. PCR with primers specific for *gar* and GFP (Table S12) were used to control success of conjugation.

To test if *gar* is located on a conjugative plasmid, we performed conjugation experiments with *E. coli* or *P. putida* as recipient. Only *P. aeruginosa* colonies grew on the selective plates, suggesting a chromosomal location of *gar*.

Table S12: Primer pairs specific to *gar* and GFP.

**Primer**	**Sequence 5' → 3'**
GFPaphA3-373	CTGTCGACACAATCTGCCCT
GFPaphA3-952	CCACATCGGCCAGATCGTTA
gar_for	ATGATTATTCTGCTTAATGGACC
gar_rev	CTAATGAAGTCCAAGCAACG

Presence and context of *gar* in metagenomes

The abundance of *gar* was searched in 1251 public metagenomic datasets (Additional file 2). Diamond [10] (v0.9.24.125) was used to map the reads to the reference protein with 100 % identity and an ORF-length greater than 20 amino acids.

To study the genetic context around the novel ARG, short reads from selected metagenomic datasets were mapped to the reference sequences from *P. aeruginosa* 105MG (AJ786649.2$_{3796:4495}$) and *Luteimonas* sp. 83-4 (CP029556.1$_{534872\text{-}536047}$) using Bowtie 2 [16] (v2.2.9). Integron attachment sites were detected by identifying marginal paired-end reads using the Tablet software [17] (v1.19.05).

Phylogenetic tree

Proteins related to GAR were selected through three iterations of PSI-BLAST [18] (-max_target_seq 1000) on NCBI's non-redundant protein database. Four proteins among the 1000 closest related were annotated as tunicamycin resistance proteins. We decided to include additionally the two published tunicamycin resistance proteins TmrB [19] (WP_003246258.1) and TmrD [20] (WP_010888058.1), because only their resistance against tunicamycin was experimentally proven. The retrieved proteins along with GAR and all aminoglycoside resistance proteins from ResFinder (protein accession numbers listed in Additional file 3) were aligned using MAFFT [21] (v7.310; --maxiterate 1000 --localpair). The phylogenetic tree was calculated by FastTree [22] (v2.1.9) using maximum likelihood algorithm, Jones-Taylor-Thornton model, with 1000 times bootstrap. The full version of the tree is available in Additional file 4. The Interactive Tree Of Life (iTOL v4) online tool [23] (https://itol.embl.de) was used to prepare the phylogenetic tree for display.

Protein model

The I-TASSER server for protein structure and function prediction was used to create models of GAR [24-26]. The model with the highest confidence score (C-score: -0.21) is shown. DeepView / Swiss-PdbViewer [27] (v4.1.0) was used to create the ribbon presentation.

References

1. Giske CG, Libisch B, Colinon C, Scoulica E, Pagani L, Füzi M, et al. Establishing clonal relationships between VIM-1-like metallo-beta-lactamase-producing Pseudomonas aeruginosa strains from four European countries by multilocus sequence typing. J Clin Microbiol. 2006;44:4309-15. doi: 10.1128/JCM.00817-06.
2. Razavi M, Marathe NP, Gillings MR, Flach C-F, Kristiansson E, Joakim Larsson DG. Discovery of the fourth mobile sulfonamide resistance gene. Microbiome. 2017;5:160. doi: 10.1186/s40168-017-0379-y.
3. Forsberg KJ, Patel S, Gibson MK, Lauber CL, Knight R, Fierer N, et al. Bacterial phylogeny structures soil resistomes across habitats. Nature. 2014;509:612-6. doi: 10.1038/nature13377.
4. Galani I, Kontopidou F, Souli M, Rekatsina P-D, Koratzanis E, Deliolanis J, et al. Colistin susceptibility testing by Etest and disk diffusion methods. Int J Antimicrob Agents. 2008;31:434-9. doi: 10.1016/j.ijantimicag.2008.01.011.
5. Nordmann P, Jayol A, Poirel L. A Universal Culture Medium for Screening Polymyxin-Resistant Gram-Negative Isolates. J Clin Microbiol. 2016;54:1395-9. doi: 10.1128/JCM.00446-16.
6. Allen HK, Moe LA, Rodbumrer J, Gaarder A, Handelsman J. Functional metagenomics reveals diverse β-lactamases in a remote Alaskan soil. ISME J. 2009;3:243-51. doi: 10.1038/ismej.2008.86.
7. Wang S, Gao X, Gao Y, Li Y, Cao M, Xi Z, et al. Tetracycline Resistance Genes Identified from Distinct Soil Environments in China by Functional Metagenomics. Front Microbiol. 2017;8:1406. doi: 10.3389/fmicb.2017.01406.
8. Hyatt D, Chen G-L, Locascio PF, Land ML, Larimer FW, Hauser LJ. Prodigal: prokaryotic gene recognition and translation initiation site identification. BMC Bioinformatics. 2010;11:119. doi: 10.1186/1471-2105-11-119.
9. Zankari E, Hasman H, Cosentino S, Vestergaard M, Rasmussen S, Lund O, et al. Identification of acquired antimicrobial resistance genes. J Antimicrob Chemother. 2012;67:2640-4. doi: 10.1093/jac/dks261.
10. Buchfink B, Xie C, Huson DH. Fast and sensitive protein alignment using DIAMOND. Nat Methods. 2015;12:59-60. doi: 10.1038/nmeth.3176.
11. CLSI. Methods for dilution antimicrobial susceptibility tests for bacteria that grow aerobically: M07-A9; approved standard. 9. ed. Wayne, PA 19087 USA: Committee for Clinical Laboratory Standards Institute; 2012.
12. Schuurmans JM, Nuri Hayali AS, Koenders BB, ter Kuile BH. Variations in MIC value caused by differences in experimental protocol. J Microbiol Methods. 2009;79:44-7. doi: 10.1016/j.mimet.2009.07.017.
13. Bankevich A, Nurk S, Antipov D, Gurevich AA, Dvorkin M, Kulikov AS, et al. SPAdes: a new genome assembly algorithm and its applications to single-cell sequencing. J Comput Biol. 2012;19:455-77. doi: 10.1089/cmb.2012.0021.
14. Wick RR, Schultz MB, Zobel J, Holt KE. Bandage: interactive visualization of de novo genome assemblies. Bioinformatics. 2015;31:3350-2. doi: 10.1093/bioinformatics/btv383.
15. Jolley KA, Bray JE, Maiden MCJ. Open-access bacterial population genomics: BIGSdb software, the PubMLST.org website and their applications. Wellcome Open Res. 2018;3:124. doi: 10.12688/wellcomeopenres.14826.1.
16. Langmead B, Salzberg SL. Fast gapped-read alignment with Bowtie 2. Nat Methods. 2012;9:357-9. doi: 10.1038/nmeth.1923.
17. Milne I, Bayer M, Cardle L, Shaw P, Stephen G, Wright F, et al. Tablet--next generation sequence assembly visualization. Bioinformatics. 2010;26:401-2. doi: 10.1093/bioinformatics/btp666.
18. Altschul SF, Madden TL, Schäffer AA, Zhang J, Zhang Z, Miller W, et al. Gapped BLAST and PSI-BLAST: a new generation of protein database search programs. Nucleic Acids Res. 1997;25:3389-402. doi: 10.1093/nar/25.17.3389.

19. Noda Y, Yoda K, Takatsuki A, Yamasaki M. TmrB protein, responsible for tunicamycin resistance of Bacillus subtilis, is a novel ATP-binding membrane protein. J Bacteriol. 1992;174:4302-7. doi: 10.1128/jb.174.13.4302-4307.1992.
20. Kapp U, Macedo S, Hall DR, Leiros I, McSweeney SM, Mitchell E. Structure of Deinococcus radiodurans tunicamycin-resistance protein (TmrD), a phosphotransferase. Acta Cryst. 2008;F64:479-86. doi: 10.1107/S1744309108011822.
21. Katoh K, Standley DM. MAFFT multiple sequence alignment software version 7: improvements in performance and usability. Mol Biol Evol. 2013;30:772-80. doi: 10.1093/molbev/mst010.
22. Price MN, Dehal PS, Arkin AP. FastTree 2 - approximately maximum-likelihood trees for large alignments. PLoS ONE. 2010;5:e9490. doi: 10.1371/journal.pone.0009490.
23. Letunic I, Bork P. Interactive Tree Of Life (iTOL) v4: recent updates and new developments. Nucleic Acids Res. 2019;47:W256-W9. doi: 10.1093/nar/gkz239.
24. Roy A, Kucukural A, Zhang Y. I-TASSER: a unified platform for automated protein structure and function prediction. Nat Protoc. 2010;5:725-38. doi: 10.1038/nprot.2010.5.
25. Yang J, Yan R, Roy A, Xu D, Poisson J, Zhang Y. The I-TASSER Suite: protein structure and function prediction. Nat Methods. 2015;12:7-8. doi: 10.1038/nmeth.3213.
26. Zhang Y. I-TASSER server for protein 3D structure prediction. BMC Bioinformatics. 2008;9:40. doi: 10.1186/1471-2105-9-40.
27. Guex N, Peitsch MC. SWISS-MODEL and the Swiss-Pdb Viewer: An environment for comparative protein modeling. ELECTROPHORESIS. 1997;18:2714-23. doi: 10.1002/elps.1150181505.
